# In search of a composite biomarker for chronic pain by way of EEG and machine learning: where do we currently stand?

**DOI:** 10.3389/fnins.2023.1186418

**Published:** 2023-06-14

**Authors:** Mika M. Rockholt, George Kenefati, Lisa V. Doan, Zhe Sage Chen, Jing Wang

**Affiliations:** ^1^Department of Anesthesiology, Perioperative Care and Pain Management, New York University Grossman School of Medicine, New York, NY, United States; ^2^Department of Psychiatry, New York University Grossman School of Medicine, New York, NY, United States; ^3^Department of Neuroscience & Physiology, Neuroscience Institute, New York University Grossman School of Medicine, New York, NY, United States; ^4^Department of Biomedical Engineering, New York University Tandon School of Engineering, Brooklyn, NY, United States

**Keywords:** chronic pain, biomarkers, neurobiomarkers, EEG, machine learning, neurophysiology, biomarker, composite biomarker

## Abstract

Machine learning is becoming an increasingly common component of routine data analyses in clinical research. The past decade in pain research has witnessed great advances in human neuroimaging and machine learning. With each finding, the pain research community takes one step closer to uncovering fundamental mechanisms underlying chronic pain and at the same time proposing neurophysiological biomarkers. However, it remains challenging to fully understand chronic pain due to its multidimensional representations within the brain. By utilizing cost-effective and non-invasive imaging techniques such as electroencephalography (EEG) and analyzing the resulting data with advanced analytic methods, we have the opportunity to better understand and identify specific neural mechanisms associated with the processing and perception of chronic pain. This narrative literature review summarizes studies from the last decade describing the utility of EEG as a potential biomarker for chronic pain by synergizing clinical and computational perspectives.

## 1. Introduction

Pain is a complex and multi-dimensional process resulting from dynamic interactions of neural processes. It includes sensory-discriminative, affective-emotional, and cognitive-evaluative components (Rainville et al., 1997; Price, 2000; Perl, 2007; Sun et al., 2021). All pain initiates as acute pain but can become maladaptive and persist into a chronic phase (Gan et al., 2014; Chapman and Vierck, 2017; Gan, 2017). Numerous studies have demonstrated clear evidence that chronic pain continues to be a global public health issue, with an estimated prevalence of around 30% in adults (Johannes et al., 2010; Gardner and Sachdeva, 2019; Yong et al., 2022; Zimmer et al., 2022). The condition is not only known to significantly reduce quality of life (Yong et al., 2022) but is also associated with long-term disability; this typically requires multimodal treatment approaches, commonly results in reliance on opioid analgesics, and contributes to the opioid epidemic (Kehlet et al., 2006; Ataoğlu et al., 2013; Ladha et al., 2016; Gan, 2017; Schuchat et al., 2017; Hollmann et al., 2019). Hence, advances in pain research are urgently needed to address these healthcare issues.

By observing the brain activity that occurs during pain and trying to decode its underlying mechanism, it is believed that these pathways could be targeted earlier and more precisely, preventing pain from chronification, and thus reducing the consumption of addictive analgesics (Mouraux and Iannetti, 2018).

Over the last 20 years, advancing research has contributed to an increased understanding of the spinal, peripheral, and cortical mechanisms of pain (Apkarian et al., 2005; Rosa and Seymour, 2014; Tu et al., 2016; Ploner et al., 2017; Mouraux and Iannetti, 2018). In contrast to other sensory perceptions that are associated with a specific sensory cortex, a specific “pain cortex” associated with pain perception does not exist (Kucyi and Davis, 2015). Instead, it is the result of an activation of a distributed network of cortical and subcortical areas (Besson, 1999; Sawamoto et al., 2000; Mouraux and Iannetti, 2018; Liberati et al., 2020; Chen et al., 2022). Because of its complex nature, further research is required to better understand the mechanisms behind pain and to propose an adequate biomarker for chronic pain in particular (Tracey et al., 2019; Chen, 2021).

Studies using modern neuroimaging techniques such as functional magnetic resonance imaging (fMRI) and positron emission tomography have identified brain regions involved in sensory processing of acute pain. These brain regions include the primary somatosensory cortex (S1), anterior cingulate cortex (ACC), and insular cortex (Isnard et al., 2011; Duerden and Albanese, 2013; Vierck et al., 2013; Boccard et al., 2014; Mouraux and Iannetti, 2018; Van Der Miesen et al., 2019; Lamichhane et al., 2021; Sun et al., 2021). These techniques have limited temporal resolution, making it difficult to capture the dynamic nature of pain perception and experience (Wager et al., 2013). Therefore, some study groups have shifted focus to explore less invasive and more cost-effective alternatives with a higher temporal resolution, such as electroencephalography (EEG) (Pinheiro et al., 2016; Ploner et al., 2017; Levitt and Saab, 2019; Van Der Miesen et al., 2019; Xu and Huang, 2020; Millard et al., 2022; Chowdhury et al., 2023).

With an increasing number of human neuroimaging studies investigating the mechanism of pain, the field is steadily moving toward the development of a viable biomarker for pain (Furman et al., 2018; Seminowicz et al., 2018; Furman et al., 2019; Seminowicz et al., 2019; Furman et al., 2020). For instance, modern source localization techniques have substantially improved anatomical precision for EEG studies to enable circuit-level analysis, further facilitating the potential of biomarker development (Ferracuti et al., 1994; Le Pera et al., 2000; Chang et al., 2001; Seminowicz et al., 2019; Furman et al., 2020; Sun et al., 2021; Chowdhury et al., 2023). Recent reports have indicated that enhanced nociceptive response in EEG is manifested as abnormally elevated power in theta and gamma oscillations, suggesting that EEG could potentially predict the presence of pain and analgesic response (Babiloni et al., 2002; Wang et al., 2011; Schulz et al., 2012a, b; Rouleau et al., 2015; Peng and Tang, 2016; Taesler and Rose, 2016; Martel et al., 2017; Fallon et al., 2018; Tan et al., 2021). This provides further support for the feasibility of an EEG-based biomarker (Zhang et al., 2017; Dinh et al., 2019; May et al., 2019; Zis et al., 2022).

By applying machine learning (ML) to analyze functional brain imaging data such as EEG, we now have the capability to better identify response features to a given experiment—or stimulus, as well as to predict subjective perception and response to the same experiment (Hu and Iannetti, 2016; Lötsch et al., 2017; Fernandez Rojas et al., 2019). Hence, ML is a promising tool for the future development of biomarkers for chronic pain (Lamichhane et al., 2021; Harland et al., 2022). Recently, numerous studies have presented findings with considerable accuracy, working toward developing algorithms with improved generalizability and interpretability (Harland et al., 2022; Mari et al., 2022). The number of studies coupling ML algorithms with subjective reports on pain perception is increasing, and there is an impetus for data-sharing and collaboration within the pain research community to improve the sensitivity and efficacy of biomarker developmental methods (Van Der Miesen et al., 2019). While obstacles remain, it is clear that “decisions based on neural data will only be as good as the science behind them” (Hu and Iannetti, 2016). Hence, for the science to get better, it is imperative that we validate the results of past studies, identify the state-of-the-art methods, and provide updates on ongoing studies.

Today, the probability of presenting a non-composite, single biomarker capturing “pain” in its entirety is increasingly appearing unlikely (Tracey et al., 2019). It remains difficult to identify a standard way to qualify brain responses as specifically pain responses, especially without a dedicated pain cortex. However, by further exploring advanced analytical tools like neural networks, artificial intelligence, and machine-learning algorithms, it may be possible to combine multiple objective biomarkers into one composite pain biomarker (Tracey, 2021). Such approach could expedite success in understanding the mechanisms for pain as well as providing clinically relevant biomarkers (Baskin et al., 2016; Su et al., 2019; Eldabe et al., 2022).

To support the field in future research, we have conducted a narrative literature review by combining the following search terms: “electroencephalography” and “chronic pain” and “machine learning” using PubMed (including MEDLINE), Ovid (including EMBASE), Web of Science and the Cochrane Library. While several review articles have appeared in the literature (Reckziegel et al., 2019; Van Der Miesen et al., 2019; Mari et al., 2022; Zebhauser et al., 2023), our review focuses on studies published in the last decade (from 2012 to 2023), describing the practical utility of combining physiological data, EEG, and ML to study the mechanisms of chronic pain. Specifically, our review aims to appraise the role and potential utility of EEG as a biomarker for chronic pain. Hence, this review discusses a limited cohort and does not cover the entire breadth of publications in the pain research field. Nonetheless, we show how different computational methods and ML algorithms can help in the discovery of EEG biomarkers for chronic pain. We also discuss the future utility of today’s cutting-edge methods and how we can incorporate further analyses and neurophysiological data into an integrated biomarker model. Lastly, we discuss challenges in the chronic pain research field and offer insight on potential future directions.

## 2. The benefits of EEG for biomarker studies on chronic pain

### 2.1. Measurement of pain-related brain activity

Behavior arising from the experience of pain is seldom obscure, often manifested physically in the form of facial expressions, changes in body language, or changes in mood (Dansie and Turk, 2013). Thus, the presence of pain can be observed and measured – to a certain extent. Pain perception is a highly subjective experience (Kinnealey and Fuiek, 1999). Pain does not only vary between subjects but can vary in the same subject across time. For this reason, a number of pain assessments are available for the quantification of pain.

#### 2.1.1. Bottom-up measurements based on pain-induced behavior

The current standard for self-reported pain intensity is assessed using the visual analog scale (VAS, typical range: 0–100 mm) (Boonstra et al., 2008) or the numeric rating scale (NRS, range: 0–10) (Haefeli and Elfering, 2006). Although these behavioral scales offer a degree of standardization across the population, individuals can still exhibit considerable and unpredictable variability in painful percepts in response to the same nociceptive stimulus (Quiton and Greenspan, 2008). Furthermore, self-reported pain intensity has been shown to at times correlate poorly with the stimulus intensity in experimental studies (Nickel et al., 2017). The mechanisms underlying such within-subject and between-subject variability in experimental pain remain insufficiently understood. The objective of top-down measurements of pain experience is to identify neural indicators that explain such perceptual variability in all types of pain (Hu and Iannetti, 2016).

#### 2.1.2. Top-down measurements based on brain activity

A wide variety of neuroimaging techniques are used to record brain signals, at varying levels of invasiveness and across a range of spatial and temporal resolutions. For instance, microelectrode implants can record neural spikes from a single neuron or local field potentials from a small population of neurons, though at the cost of invasiveness and highly rapid time courses (Einevoll et al., 2013; Nurse et al., 2016; Merk et al., 2022). At the other end of the spectrum, whole brain imaging technologies such as fMRI can noninvasively image blood-oxygen dynamics as a proxy to region-specific activity, yet with relatively low temporal resolution and mid-range spatial resolution (Logothetis, 2008; Power et al., 2017; Woo et al., 2017; Siddiqi et al., 2022). In this review, we focus solely on EEG which measures extracellular current but at a larger spatial scale, reflecting the activity of hundreds of millions of neurons (Jackson and Bolger, 2014). EEG is noninvasive: the signal is recorded from an array of surface electrodes placed on a subject’s scalp, at a microvolt (μV) scale (Rosa and Seymour, 2014). Despite anatomical impedances, such as the presence of hair and variations in skull conductivity, EEG electrodes are capable of detecting the electrical activity of similarly oriented groups of cerebral cortical neurons near the scalp. The majority of the electrical activity sensed by scalp electrodes represents the summation of the inhibitory or excitatory postsynaptic potentials from thousands of pyramidal cells near each electrode (Britton et al., 2016). This trait enables researchers and clinicians a view into the cortical activity of the brain with low cost and effort.

EEG can be recorded using one of two methods: resting-state EEG (rs-EEG) or stimulus-evoked EEG. rs-EEG is recorded while a participant is awake but not engaged in any specific task. Though rs-EEG studies do not typically include stimuli, they may be used to evaluate the functional activity of the brain before and after a treatment or intervention, as well as to study chronic behavioral or pathological conditions such as chronic pain. Stimulus-evoked EEG, in contrast, is recorded in response to a specific stimulus. Typically, participants are subject to a set of repeated stimuli to study the dynamics of one or more regions involved in responding to said stimulus. Thus, stimulus-evoked EEG provides temporally specific information in a dynamic behavioral context, and it also offers insights into how an underlying disease condition such as chronic pain can alter temporal sensory processing. Both techniques are equally important, even as they enable fundamentally different approaches to studying chronic pain which are discussed in the next section.

### 2.2. Advantages and disadvantages of EEG for chronic pain studies

Despite the advances in pain research using other neuroimaging techniques, such as fMRI and PET, numerous studies have described the advantages of leveraging the non-invasiveness of EEG as a potential path to a biomarker for pain (Ploner et al., 2017; Liberati et al., 2018; Mouraux and Iannetti, 2018; May et al., 2019; Sun et al., 2021; Tracey, 2021; Vuckovic et al., 2022).

First, because of its high temporal resolution, it allows us to assess the oscillatory activity of neural pain processing. High temporal resolution is critical for understanding pain since it is a highly dynamic process (Schulz et al., 2012a,b). At the same time, correlating oscillatory activities across different brain areas enables us to detect specific brain areas associated with chronic pain. As such, in chronic pain studies, scalp EEG recordings present spontaneous synchronized postsynaptic neuronal activity of the brain cortex (Huber et al., 2006; Stern et al., 2006; Jobert et al., 2012; Mouraux and Iannetti, 2018).

A second advantage of EEG in the study of pain is that it is portable, easy to perform, well tolerated by patients, and more cost-effective than other neuroimaging modalities (Katsigiannis and Ramzan, 2017; Krigolson et al., 2017; Mussigmann et al., 2022). The ease of placement and mobility of EEG systems allows for continuous recordings of primary cortical activities in clinical settings, enabling the potential to develop a variety of biomarkers for chronic pain from a single modality (Byrom et al., 2018; Xu and Huang, 2020).

Meanwhile, a number of potential obstacles in using EEG to assess chronic pain should also be considered (Tracey, 2021). First, there are some limitations seen in past studies, where most have analyzed potentials from rs-EEG (as presented in Table 1). These potentials provide a basis for further analyses, allowing us to explore the dynamic of neural circuits involved in pain processing. The use of rs-EEG alone in understanding pain, however, can become problematic, since rs-EEG potentials may be confounded by other brain processes. As suggested by Hansen et al. (2017) studying evoked EEG potentials allows for a better understanding of the mechanism underlying nociception, which is especially important when studying a complex condition such as chronic pain (Cao et al., 2020). Thus, when studying chronic pain, rs-EEG can provide us with insights into baseline differences, whereas evoked EEG potentials can contain information about acute changes in neural pain processing, which can be more informative (Plaghki and Mouraux, 2005). Regardless of the types for potentials studied, uncertainties around whether recorded EEG responses are directly related to pain still exist, prompting the need for further research (Mouraux and Iannetti, 2018).

**Table 1:** Summary of recent studies utilizing EEG as a potential biomarker for chronic pain (review articles are excluded from this summary).

Author	Subjects	Chronic pain	EEG State^a^	Feature/Machine learning analysis	Findings (significance, *p*-value; accuracy, %)	Biomarker type
2012						
Graversen et al.	N_pain_ = 31	Pancreatitis	rs-EEG	PSD, SVM	Pregabalin: decreased pain / increased theta power (*p* = 0.03; 85.7%)	Monitoring
Mendonça-de-Souza et al.	N_pain_ = 11 N_healthy_ = 7	Migraine	rs-EEG eEEG	FBP	Differences in cortical coherence before and after photic stimulation (*p* < 0.05)	Diagnostic, prognostic
Schmidt et al.	N_pain_ = 37 N_healthy_ = 37	Low Back Pain	rs-EEG	Peak frequency, PSD	No significant findings observed (*p* > 0.05)	N/A
2013						
Jensen et al.	N_pain_ = 10	Any	rs-EEG eEEG	PSD	Neurofeedback treatment: decreased pain/decreased theta (*p* = 0.004), increased alpha power (*p* = 0.002)	Monitoring
van den Broeke et al.	N_pain_ = 8 N_healthy_ = 11	Post Mastectomy	rs-EEG eEEG	CoG	Enhanced alpha activity (7–13 Hz) in parietal and occipital cortices (*p* < 0.05)	Diagnostic
De Vries et al.	N_pain_ = 16 N_healthy_ = 16	Pancreatitis	rs-EEG	Peak alpha frequency	Decreased peak alpha frequencies (*p* = 0.049)	Diagnostic
2014						
Vuckovic et al.	N_pain_ = 10 N_healthy_ = 20	Central Neuropathic Pain	rs-EEG eEEG	PSD	Increased ERD in theta, alpha and beta bands (16–24 Hz) (*p* = 0.0085)	Diagnostic^b^
Sufianov et al.	N_pain_ = 30 N_healthy_ = 10	FBSS	rs-EEG	PSD	Differences in peak alpha frequency, beta and theta power after epidural spinal cord stimulation (*p* < 0.05)	Diagnostic, monitoring
2015						
Navarro López et al.	N_pain_ = 13 N_healthy_ = 13	Fibromyalgia	rs-EEG	PSD	Decreased theta and absolute alpha power, increased beta power (*p* < 0.05)	Diagnostic
Schmidt et al.	N_pain_ = 21	Back Pain	rs-EEG	Peak frequency, peak power, CoG	Mindfulness-based stress-reduction: no significance	Monitoring
2016						
González-Roldán et al.	N_pain_ = 20 N_healthy_ = 18	Fibromyalgia	rs-EEG	PSD, sLORETA, current source distribution	Negative correlation between delta band power and pain duration (*p* = 0.026). Increased theta power (*p = 0.04*), reduced alpha response (*p = 0.017*). Significant changes in beta bands (*p < 0.05*)	Diagnostic
Meneses et al.	N_pain_ = 21 N_healthy_ = 21	Rheumatoid Arthritis	rs-EEG	PSD	Increased absolute and relative alpha power densities (*p* < 0.05)	Diagnostic
2017						
Gram et al.	N_pain_ = 81	Hip Pain	rs-EEG eEEG	PSD, functional connectivity, SVM	Frontal delta power and functional connectivity features influence post-operative treatment response (65%)	Predictive
Camfferman et al.	N_pain_ = 103	Any	rs-EEG	Spectral band power	Negative association between alpha power and pain intensity in frontal and parietal areas (*p* < 0.01)	Diagnostic
Thibaut et al.	N_pain_ = 5 N_healthy_ = 47	Pancreatitis	rs-EEG	PSD	Transcranial electrical stimulation (tPCS/tDCS): differences in alpha, beta (*p* < 0.05), and theta (*p* ≤ 0.002) band power.	Diagnostic, monitoring
Prinsloo et al.	N_pain_ = 62	Peripheral Neuropathy	rs-EEG eEEG	PSD	Neurofeedback therapy: increased alpha power (*p* = 0.021) and decreased beta power (*p =* 0.021)	Monitoring
2018						
Cao et al.	N_pain_ = 40 N_healthy_ = 40	Migraine	rs-EEG	Inherent fuzzy entropy; SVM	Changes in EEG patterns between phases of an ongoing migraine attack (76%)	Prognostic
Di Pietro et al.	N_pain_ = 20 N_healthy_ = 20	Trigeminal Neuropathy	rs-EEG	PSD	Differences in theta (*p* = 0.04), beta and low alpha ranges (*p* = 0.03)	Diagnostic
Fallon et al.	N_pain_ = 19 N_healthy_ = 18	Fibromyalgia	rs-EEG eEEG	sLORETA	Increased theta power in prefrontal cortex, anterior cingulate cortex and DLPFC (*p* < 0.05)	Diagnostic
Vanneste et al.	N_pain_ = 78 N_healthy_ = 264	Neuropathic Pain	rs-EEG	sLORETA; SVM	Thalamocortical dysrhythmia may serve as a mechanism underlying pain (92.5%)	Diagnostic
Vuckovic et al.	N_pain_ = 11 N_healthy_ = 31	Central Neuropathic Pain	rs-EEG	Band power analysis; SVM	Transferable learning classifier could detect patients developing chronic neuropathic pain based on alpha band features (> 85%)	Prognostic^c^
Prichep et al.	N_pain_ = 77 N_healthy_ = 77	Any	rs-EEG	sLORETA	Overactivation of the cingulate gyrus, insula, parietal lobule, the thalamus and the DLPFC (90%)	Diagnostic, monitoring
Zhou et al.	N_pain_ = 14 N_healthy_ = 14	Postherpetic Neuralgia	rs-EEG	PSD, Phase-amplitude coupling	Increased gamma power (prefrontal and cerebellar areas); positive correlation with pain intensity (*p* < 0.05)	Diagnostic, monitoring
2019						
Dinh et al.	N_pain_ = 101 N_healthy_ = 84	Any	rs-EEG	Functional connectivity, SVM	Increased connectivity: theta and gamma frequencies frontally, global network reorganization (57%)	Diagnostic
Ahn et al.	N_pain_ = 20	Low Back Pain	rs-EEG	Alpha band power	Transcranial stimulation (tACS): increased alpha power in somatosensory regions indicating pain relief (*p* < 0.05)	Diagnostic, monitoring
Ferdek et al.	N_pain_ = 20 N_healthy_ = 17	Endometriosis	rs-EEG eEEG	Directed transfer function connectivity	Increased beta connectivity: left DLPFC, somatosensory, orbitofrontal and right temporal cortex (*p* < 0.05)	Diagnostic
Villafaina et al.	N_pain_ = 31 N_healthy_ = 31	Fibromyalgia	rs-EEG	Spectral power	Reduced alpha-2 (11–12 Hz) power with negative VAS pain score correlations (*p* < 0.05)	Diagnostic
Yüksel et al.	N_pain_ = 42 N_healthy_ = 21	Fibromyalgia	rs-EEG	PSD	Changes in anterior delta, theta, alpha and beta power with TENS and acupuncture (*p* < 0.05)	Diagnostic, Monitoring
2020						
Baroni et al.	N_pain_ = 24 N_healthy_ = 24	Orofacial Pain	rs-EEG eEEG	Z-scored PSD	Decreased alpha and increased gamma activity in central and frontal regions (*p < 0.05*)	Diagnostic
de Melo et al.	N_pain_ = 31	Fibromyalgia	rs-EEG	PSD	Transcranial stimulation therapy (tDCS); differences in frontal and parietal alpha-2 band power bands (*p* < 0.05)	Diagnostic, monitoring
Levitt et al.	N_pain_ = 37 N_healthy_ = 20	Low Back Pain	rs-EEG	PSD, Phase-amplitude coupling, SVM	Differences in low-gamma power (42–43 Hz) (82.5%)	Diagnostic
González-Villar et al.	N_pain_ = 43 N_healthy_ = 51	Fibromyalgia	rs-EEG	Temporal-concatenation group connectivity, PLI	Increased beta connectivity with shorter microstate occurrence/functional connectivity (*p* < 0.05)	Diagnostic
Telkes et al.	N_pain_ = 9	Any	rs-EEG eEEG^d^	PSD	Differences in alpha-theta spectral dynamics in prefrontal, frontal and S1 cortices (*p* < 0.05), increasing alpha band power	Monitoring
Uygur-Kucukseymen et al.	N_pain_ = 26	Fibromyalgia	rs-EEG eEEG	PSD, ERD	Reduced alpha power in frontal, central and parietal areas, reduced beta power in central areas (*p* < 0.05), smaller ERD responses in theta and delta bands (*p* < 0.05)	Diagnostic, Predictive
2021						
Patel et al.	N_pain_ = 4 N_healthy_ = 4	Any	rs-EEG eEEG	Alpha power	Transition probabilities from low to high alpha state after alpha-neurofeedback therapy (*p* < 0.05)	Monitoring
Barbosa-Torres et al.	N_pain_ = 37	Fibromyalgia	rs-EEG eEEG	Spectral band amplitude	Neurofeedback therapy: changes in theta wave ratio before and after treatment (*p* < 0.005)	Monitoring
Bernardi et al.	N_pain_ = 15	Fibromyalgia	rs-EEG	Spectral power	Transcranial stimulation (tACS): increased alpha power (*p* = 0.024) and reduced pain symptoms (*p* < 0.05)	Monitoring
Buchanan et al.	N_pain_ = 57 N_healthy_ = 54	Post-Concussive Syndrome	rs-EEG	PSD, SVM	Increased delta and theta power (87.6%)	Diagnostic^e^
Feng et al.	N_pain_ = 27	Low Back Pain	rs-EEG	Alpha band power	Negative correlation between central alpha power and pain intensity (*p =* 0.01; strongest at Cz *p* = 0:04)	Diagnostic
Jensen et al.	N_pain_ = 173	Any	rs-EEG	Spectral band power	Lower pain intensity across all intervention groups no significance in EEG differences	Predictive, Monitoring
Lee et al.	N_pain_ = 11	FBSS	rs-EEG	sLORETA source-localized spectral band power	Pain improvement with increased activity in the right anterior cingulate cortex after non-invasive painless signaling therapy (*p* < 0.05)	Monitoring
Lendaro et al.	N_pain_ = 16 N_controls_ = 10	Phantom Limb Pain	rs-EEG	CSP, C-support vector classification	Potential to discriminate between pain and no pain using broad-band (4–40 Hz) CSP features (93.7%, leave out cross-validation)	Diagnostic
Kimura et al.	N_pain_ = 23	Hip Pain	eEEG	Sub-band power spectrum, SVM	Differences among subjects with different levels of hip pain at frequencies ranging from 1 to 35 Hz (79.6%)	Monitoring
Martín-Brufau et al.	N_pain_ = 23 N_healthy_ = 23	Fibromyalgia	rs-EEG	sLORETA, spectral band power, coherence	Decreased amplitudes in theta and alpha and beta frequencies (p < 0.01), with scarce cortical interconnections for delta and beta bands and greater functional connectivity in insular and frontal regions (*p* < 0.01; 91.3–100%)	Diagnostic
May et al.	N_pain_ = 101 N_healthy_ = 88	Any	rs-EEG	Microstate analysis	Decreased presence of microstate D, potentially related to dysfunctional attentional processes (*p* < 0.002)	Diagnostic
Parker et al.	N_pain_ = 16	Neuropathic Pain	rs-EEG	Spectral band power	Dorsal root ganglia stimulation: increased frontal, central and parietal beta power (*p* < 0.003), reduced pain intensity	Monitoring
Santana et al.	N_pain_ = 22 N_healthy_ = 18	Hip Pain	rs-EEG eEEG	Motif synchronization	Impaired dynamic brain network with shorter full synchronization time in rest network and more pronounced diffuse connectivity (*p* = 0.007)	Diagnostic
Teixeira et al.	N_pain_ = 12 N_healthy_ = 10	Peripheral Neuropathic Pain	rs-EEG	Power band analysis	Changes in GABAergic lower beta oscillation (global power spectrum decrease) (*p* = 0.007)	Diagnostic
Zortea et al.	N_pain_ = 47	Fibromyalgia	rs-EEG	Average spectral power	Decreased theta and beta peak amplitudes in opioid users vs. non-opioid users (67–73% sensitivity, 62–72% specificity)	Monitoring
2022						
Wei et al.	N_pain_ = 70	Postherpetic Neuralgia	rs-EEG	Sub-band power spectral entropy, kNN	Central-parietal beta band spectral different in treatment-resistant and treatment-sensitive patients (80% ± 11.7%)	Predictive
Teel et al.	N_pain_ = 121, N_healthy_ = 39	Muskuloskeletal Pain	rs-EEG eEEG	Theta band permutation entropy; radial basis functional kernel SVM	Theta permutation entropy features distinguishes between baseline and cold pressor task conditions in chronic pain (75.6%)	Diagnostic
Teixeira et al.	N_pain_ = 30	Low Back Pain	rs-EEG eEEG	Spectral band power	Differences in EEG frequencies between pain response and higher pain over frontal, central and parietal cortices (*p* < 0.05)	Diagnostic
Topaz et al.	N_pain_ = 133, N_healthy_ = 47	Diabetic Polyneuropathy	rs-EEG	Spectral correlation by MSC; C-support vector classification	Painful diabetes polyneuropathy patients present significantly higher cortical functional connectivity in theta (*p* = 0.008) and alpha (*p* = 0.001) bands,	Diagnostic
Heitmann et al.	N_pain_ = 41	Any	rs-EEG	Peak frequencies and CoG, functional connectivity	Interdisciplinary multimodal pain treatment reduced pain and showed an increase in theta global network efficiency (*p* < 0.001)	Monitoring

Center of Gravity (CoG); Common Spatial Pattern (CSP); Dorsolateral Frontal Cortex (DLPFC); Event-related Desynchronization (ERD); Frequency Band Power (FBP); Failed Back Surgery Syndrome (FBSS); k-Nearest Neighbors Classifier (kNN); Magnitude-Squared Coherence (MSC); Phase-Lag index (PLI); Power Spectral Density (PSD); Support Vector Machine (SVM).

a Resting-state EEG (rsEEG) or evoked EEG (eEEG).

b Suggested this type of biomarker to also serves as a prognostic biomarker.

c Continuation of previous study where the same data was to propose a combined diagnostic and prognostic biomarker.

d Intraoperative continuous EEG recordings in patients undergoing surgery for implantation of spinal cord stimulator (SCS).

e Findings are suggested to have a prognostic value in the future, where they could be applied to assess treatment response and guide treatment strategies.

Second, since evoked potentials of EEG signals are usually brief in duration, the ability to establish generalizable features can be difficult (Iannetti et al., 2008). Instead, studies have suggested longer sensory stimuli exposure to capture the true nature of evoked pain perception, where the results have shown a positive correlation with gamma power changes in the medial prefrontal cortex (Schulz et al., 2015; Misra et al., 2017).

Third, scalp EEG electrodes only record compounded peripheral neuronal activity, meaning that the signal from deeper brain structures cannot be easily separated (Hallez et al., 2007). Further studies using depth electrodes, as in invasive intracranial EEG (iEEG) which are placed in the deep structures, such as the hippocampus, amygdala, and insula, could help us to understand better the complexities of chronic pain, and its affective-emotional and cognitive-evaluative components (Peyron et al., 2002; Mokhtari et al., 2019).

Overall, the use of EEG for chronic pain studies is appealing for its high temporal resolution, low cost, broad availability, and ease of data collection (Morton et al., 2016).

## 3. Extracting pain-related features from EEG data

With the advances in pain research, the field has made significant progress in improving and streamlining the analysis of EEG measurements. Following data acquisition, the first step in a neural data analysis pipeline is preprocessing. Preprocessing, including spectral filtering and artifact rejection, extracts the signals of interest while suppressing noise to maximize the signal-to-noise ratio (Hasenstab et al., 2015). Next, the preprocessed data is used to perform feature extraction, which aims to extract only the most discriminative information from a given signal (Pedroni et al., 2019).

### 3.1. Preprocessing: artifact removal

Human EEG recordings are highly susceptible to artifacts (e.g., head movement, eye blinks, and heartbeat). Extraction and removal of these components is typically accomplished by independent component analysis (ICA) (Urigüen and Garcia-Zapirain, 2015). Once the independent components have been identified, they can be analyzed and classified as either endogenous (e.g., muscular/ocular movement, cardiac activity) or exogenous artifacts (e.g., electronic device interference, electromagnetic radiation), and subsequently removed from the EEG data (Jas et al., 2017; Jiang et al., 2019).

### 3.2. Resting-state versus stimulus-evoked processing

The signal processing pipeline differs for resting-state and stimulus-evoked types of EEG data. While the activity recorded in evoked EEG can be associated with specific emotional, motor, sensory, perceptive and cognitive processes, that of rs-EEG cannot be associated with specific events; in relation, its activity is purely spontaneous. Accordingly, evoked data may consist of dozens to hundreds of repeated, seconds-long epochs (Aunon et al. 1978; Hu et al. 2019) while rs-EEG data is composed of one recording ranging from a few minutes to several hours in duration (Khanna et al., 2015; Olejarczyk et al., 2017), Therefore, evoked EEG data is accompanied by trial time-stamps, trial labels, and subject responses, while rs-EEG data may only contain sparse annotations. The differences in processing pipelines for the two data types are summarized below.

**Common resting-state processing techniques:**

- Omit trace segments with amplitude values above a set threshold for each electrode.
- Visually or programmatically omit trace segments containing movement artifacts.
- Set a single baseline as amplitude reference
- Cropped total duration.

**Common evoked signal processing techniques:**

- Programmatically drop entire epochs containing movement artifacts.
- Scale amplitude values across epochs, especially for cross-subject analyses.
- Set a single baseline as reference, or independent baseline preceding each epoch.

### 3.3. EEG feature extraction

The extraction of features from EEG data involves prior knowledge of the brain activity potentially related to pain processing (Hu and Zhang, 2019). Examples of such prior knowledge may include which brain regions are involved in pain processing, the timing and synchronization of activity—both within and between regions, and the degree of connectivity between those regions. Features commonly used in EEG studies of chronic pain can be represented in the spatial, temporal, or spectral domains (or a combination of the three) and computed from either sensor space or source space data. These features are typically analyzed for temporal dynamics more than rs-EEG and the different feature representations contribute to the investigation of pain processing from distinct yet meaningful perspectives.

#### 3.3.1. Spatial features

Extracting meaningful spatial patterns in EEG data by methods such as dimensionality reduction and pattern optimization allows for the identification of specific regions involved in pain processing. For instance, common spatial patterns is a linear algebra-based technique that works by finding the most discriminative EEG components between different classes in a given dataset, such as trials during painful stimulation versus trials without (Blankertz et al., 2008; Lu et al., 2010; Wu et al., 2014).

#### 3.3.2. Temporal and spectral features

**Figure 1:**
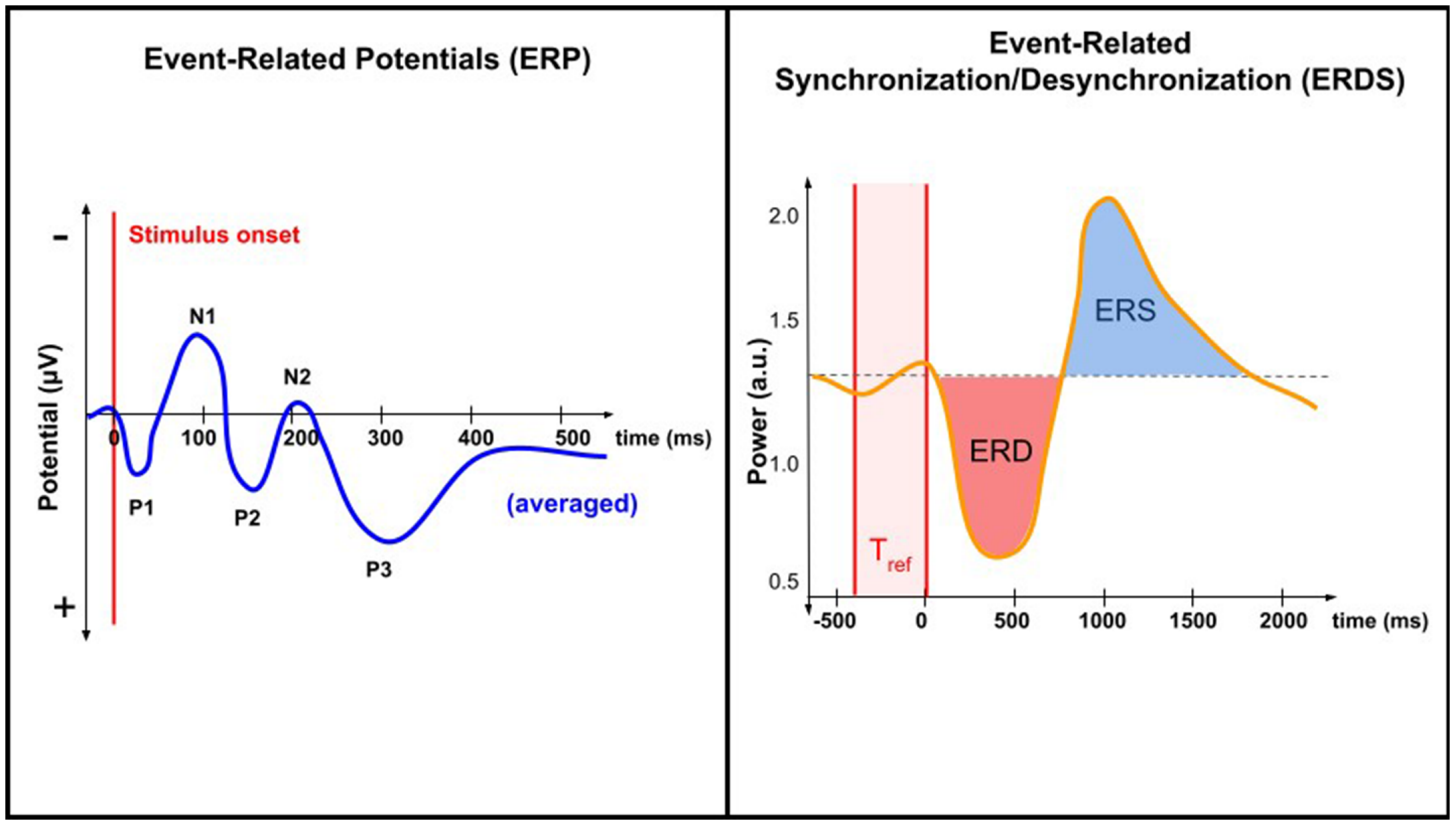
Comparing ERP and ERD/ERS analysis techniques. ERP analysis involves EEG data time-locked to a specific event. When averaged, ERPs reveal characteristic peaks and troughs (N1, P1, etc.). ERDS analysis involves quantifying changes in the power (rather than potential) of specific frequency bands in the EEG signal relative to a baseline period, T_ref_. Both techniques study changes in neural activity associated with a specific task or event, but only ERP is time-locked.

Pain processing is associated with complex temporal-spectral patterns of brain activity. Brain oscillations are patterns of synchronized electrical activity that arise from the coordinated activity of large populations of neurons; they can vary in amplitude, timing, and frequency. Features constructed from brain oscillations may take the form of power spectral density, relative power ratio, amplitude, phase coherence, and phase synchrony (Riaz et al., 2015). Pain-evoked event-related potentials (ERPs) are associated with an increase in theta band (4–8 Hz) power, also referred to as the theta-ERS (Pinheiro et al., 2016). In evoked pain, EEG studies have shown increased activity in the high-gamma band (60–100 Hz) (Ploner et al., 2017). In chronic pain, decreases in the power of the alpha band have also been observed (see Table 1) (De Vries et al., 2013). An increase or decrease in the power of a certain frequency band is referred to as non-phase-locked event-related synchronization (ERS) or event-related desynchronization (ERD), respectively (Pfurtscheller 2001; Hadjileontiadis, 2015). Figure 1 illustrates the differences between the ERP and ERD/ERS analysis techniques.

Higher-order information can also be extracted from both temporal and spectral features. One such example based on spectral features is the center of gravity (CoG). Assuming a defined region of interest (ROI) composed of either a subset of channels or a current source density distribution, CoG is defined as the frequency at which the whole EEG power within the empirically defined window is split into two equal parts, each part possessing the same overall power (Schmidt et al., 2012). Another example of a higher-order feature is entropy, also known as complexity. Based on information theory, entropy is a method for quantifying the irregularity of the EEG signal. When applied to the EEG power spectrum, entropy can measure the “peakedness” or “flatness” of the power distribution, representing the rhythmicity of the signal based on changes in the proportions of power at each frequency (Inouye et al., 1991).

#### 3.3.3. Source localization

Source localization in EEG is a method of estimating the location and intensity of current sources generated from cortical and even subcortical regions (Seeber et al., 2019). Minimum norm estimate, Low-resolution electromagnetic tomography (LORETA), and Beamforming are some examples of source localization algorithms (Chen et al., 2002; So, 2011; Michel and Brunet, 2019). Following EEG source localization with a subsequent functional connectivity analysis is commonly done (Schoffelen and Gross, 2009; Sohrabpour et al., 2016).

#### 3.3.4. Connectivity patterns

Functional connectivity (FC) analysis in EEG typically involves computing the statistical dependence or relationship between different brain regions or networks (Sakkalis, 2011). Some common examples of these algorithms include coherence, correlation, partial correlation, wavelet coherence, dynamic causal modeling (DCM), and Granger causality (Guo et al., 2020). These algorithms can provide insight into the direction and strength of connectivity between brain regions, as well as the dynamic nature of these connections. For example, FC has been used to identify long-range nociceptive information flow within the brain in chronic pain conditions (Necka et al., 2019).

## 4. Application of machine learning to EEG studies of chronic pain

### 4.1. Overview of machine learning algorithms

While the preprocessing and feature selection stages extract information from EEG data, the subsequent decoding stage utilizes the extracted features to provide insights for clinical and research applications. The application of ML facilitates tasks such as classification, detection, prediction, and risk assessment to identify meaningful features from EEG data (Müller et al., 2008; Hosseini et al., 2020). Then, after meeting certain criteria such as specificity, sensitivity, and generalizability, these features can be deemed biomarkers. In the context of chronic pain, this would allow us to identify patterns in EEG that could serve as putative neural codes for diagnosis, prognosis, monitoring, or prediction of chronic pain (Brodersen et al., 2012; Wager et al., 2013; Chen, 2021; Tracey, 2021; Harland et al., 2022).

Supervised and unsupervised ML approaches remain the most common approaches used in EEG studies of chronic pain cohorts (Alloghani et al., 2020). However, semi-supervised learning involves a small portion of labeled samples and a large number of unlabeled samples from which a model must learn and make predictions on new samples (Jia et al., 2014; She et al., 2019). Currently, the majority of published studies apply supervised learning to analyze EEG findings (Hammal and Cohn, 2012; Jenssen et al., 2021; Harland et al., 2022). By identifying spatial, temporal, or spectral features from the EEG data, one can train a parametric or nonparametric classifier on the labeled data to accomplish a certain task (Matsangidou et al., 2021). Examples of such tasks are described in Figure 1.

### 4.2. Supervised versus unsupervised learning approaches

Supervised and unsupervised learning methods differ primarily in their approaches to training, specifically in their reliance on labels (Aggarwal and Chugh, 2022). In the context of pain studies using stimulus-evoked EEG, the availability of labeled samples is dependent on many factors. For one, the collection of a sufficiently large number of trials (>100) in human subjects is time-consuming and often difficult for chronic pain patients who generally experience a heightened level of discomfort. Additionally, the selection of pain stimulus device, method, and intensity is limited and requires approval due to considerations of safety and ethics (Gatchel et al., 2016). To alleviate overfitting, both regularization and dimensionality reduction techniques are often employed. Another common concern in supervised learning is sample imbalance between classes. Under-sampling from the class with more trials is one way to alleviate the problem; though in pain studies, could lead to reducing an already low number of trials.

In a recent systematic review by Mari et al. (2022), they reviewed a total of 44 studies evaluating the effectiveness of ML algorithms on EEG data to explore the various aspects of pain. The continuous improvement of various performing models demonstrated high accuracies, ranging between 62 to 100%. These findings show that ML has the potential to predict pain outcomes, such as pain intensity, pain phenotype, and treatment response (Mari et al., 2022). The majority of the publications included were based on supervised ML methods, which are also known to present higher accuracies than their unsupervised counterparts (Hosseini et al., 2020).

Recently, Sun et al. developed an unsupervised learning method based on linear features extracted from EEG recordings to detect pain signals with a reported accuracy of 76% (Sun et al., 2021). By looking at source-localized ROIs using a state-space model, they observed that the unsupervised learning method requires fewer training trials and suggested that its performance is comparable—or perhaps better than the supervised method (Sun et al., 2021). However, this study assessed EEG signals from healthy pain-free subjects, with trials of acute evoked pain. To our knowledge, no studies describing the application of unsupervised learning methods to chronic pain data yet exist.

**Figure 2:**
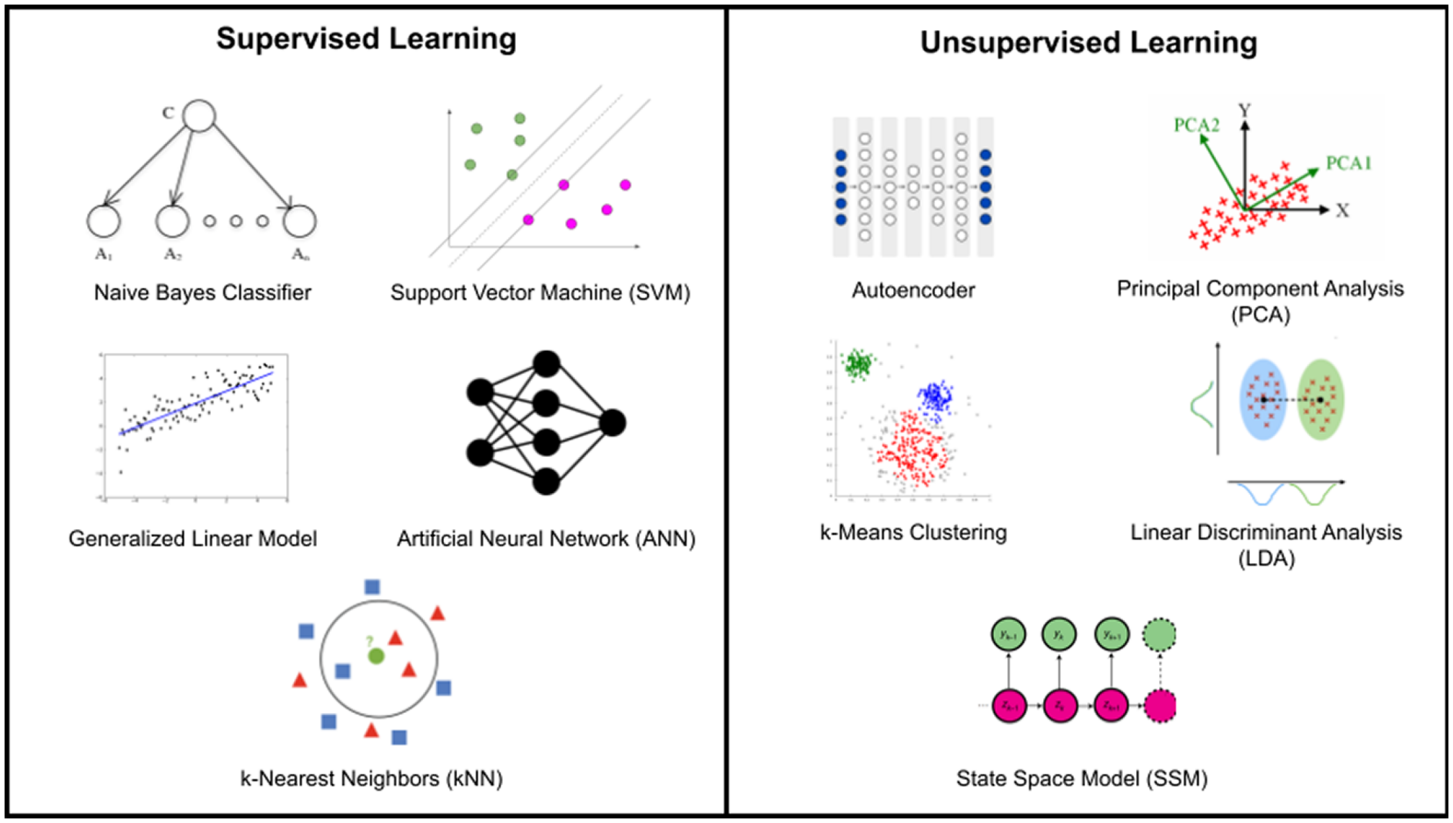
Common supervised and unsupervised classifiers applied in chronic pain research.

Unsupervised learning can alleviate the need for a large, balanced dataset of labeled samples. For example, in chronic pain research, cluster algorithms can be used when looking into pain intensities (Kragel et al., 2018). Furthermore, unsupervised learning methods have proven useful in extracting nonlinear features, making them more attractive as a decoding method in EEG pain research. However, further development is needed to demonstrate that unsupervised methods can support their generalizability with sufficiently high performance. Examples of common supervised and unsupervised classifiers employed in chronic pain research are described in Figure 2.

Semi-supervised learning approaches are worth exploring as they may offer practicality in the face of limitations: sparsely labeled data. EEG data requires administering stimuli (in the case of evoked data) or prolonged recording periods (in the case of resting-state data). For some chronic pain patients, prolonged sedentary periods can become overly uncomfortable, thereby resulting in diminishing returns with longer recordings due mostly to movement artifacts. Because semi-supervised learning requires significantly fewer labels over an entire dataset, individual recording sessions can be optimized and sped up, thereby resulting in data with higher quality and quantity. Such bottom-up approaches where the analysis step influences the data collection protocol can oftentimes lead to the best outcomes, facilitated by proper feedback.

### 4.3. Important considerations for ML in EEG studies of chronic pain

While choosing the right ML algorithm is indispensable, the importance of employing good ML practices cannot be understated. First-time users of ML may follow practices that lead to error-prone analyses, or to the illusion of successful results due to phenomena such as overfitting (model memorization of training data). By taking care in properly arranging input data, selecting an algorithm and its parameters deliberately, and appropriately evaluating model performance, one can be sure to maximize the potential in their dataset (Chicco, 2017).

Deep learning, a type of ML based on neural networks, can be highly effective at identifying nuanced pain-related features in EEG (Chen, 2021). However, deep learning requires a large number of labeled samples in training, restricting its use in the chronic pain cohort. While some studies have shown promising results in studying chronic pain with deep learning (Vuckovic et al., 2018), they are not as widespread because of the limited sample size.

## 5. Types of potential EEG biomarkers and their utility in chronic pain research

The most acknowledged definition of a biomarker is “a defined characteristic that is measured as an indicator of normal biological processes, pathogenic processes or responses to an exposure or intervention” (Group, 2016). In the context of chronic pain, a biomarker could thus serve to either confirm the presence of pain, identify the transition from one pain state to another, measure the risk of developing pain, estimate a prognosis, or predict and evaluate intervention responses (Van Der Miesen et al., 2019).

**Figure 3:**
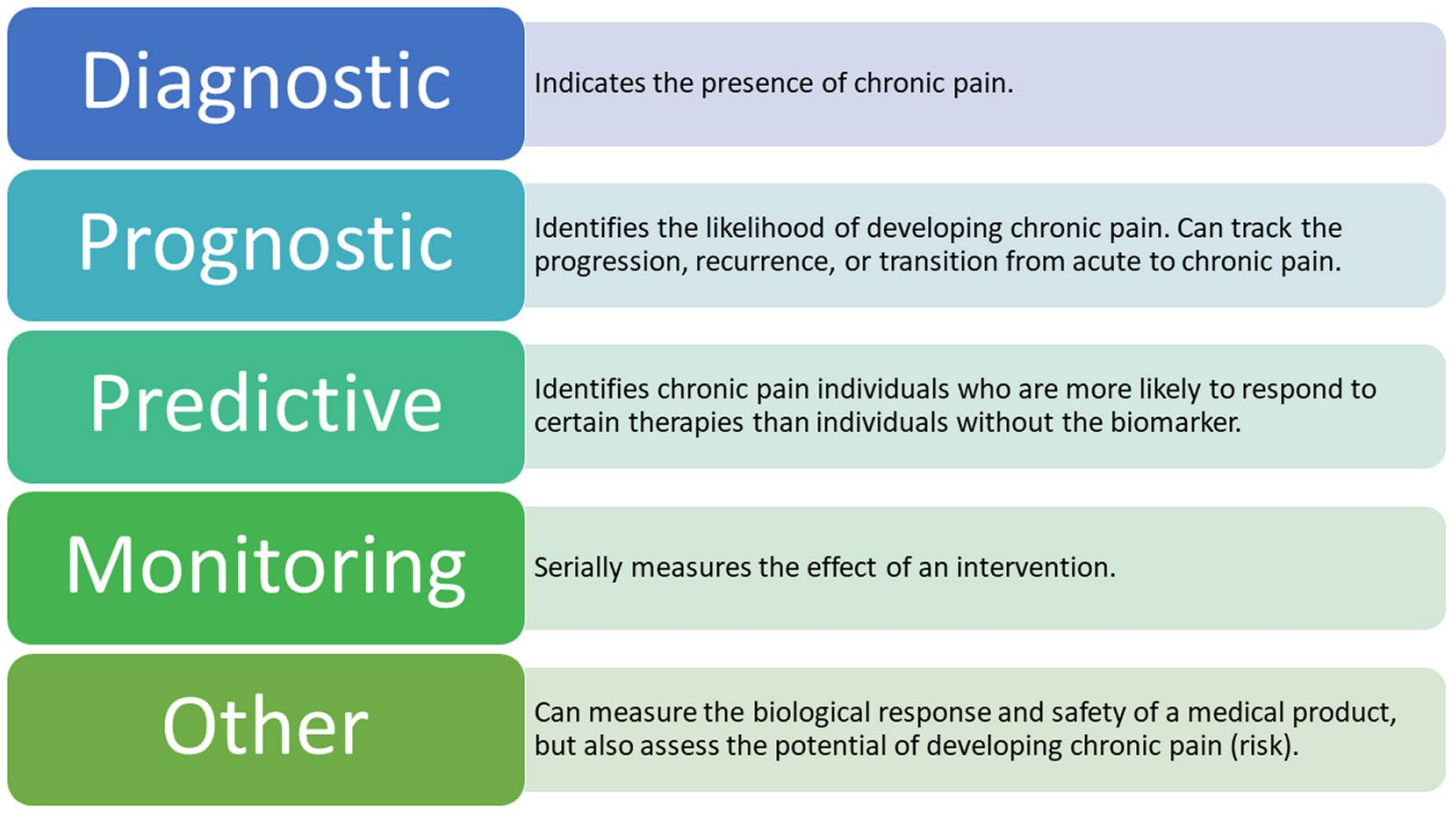
Types of Potential EEG Biomarkers for Chronic Pain. The following types of biomarkers have the potential to be clinically applicable in chronic pain management (Tracey et al., 2019; Van Der Miesen et al., 2019) A combination of these biomarkers is also a possible outcome for future research.

Clinical biomarkers can be further classified based on their presumed application and purpose (Group, 2016; Califf, 2018; Tracey et al., 2019; Van Der Miesen et al., 2019; Eldabe et al., 2022). The various types of biomarkers according to the most recent literature are presented in Figure 3. Until recently, the main objective of biomarker development has focused less on quantifying pain and more on delivering high-accuracy diagnoses and treatment algorithms, although based on neural mechanisms, rather than on symptoms (Dahlhamer et al., 2018; Gunn et al., 2020).

There are seven defined biomarker subtypes, each belonging to one of four categories associated with the development of biological biomarkers (Group, 2016; Tracey et al., 2019; Van Der Miesen et al., 2019). To date, the most applied biomarker subtypes in chronic pain research are diagnostic, prognostic, predictive, and monitoring (Davis et al., 2020). By identifying specific brain regions involved in the processing of chronic pain signals, we are indeed getting closer to decoding the presence of chronic pain (Graversen et al., 2012; Mendonça-de-Souza et al., 2012; Schmidt et al., 2012; De Vries et al., 2013; van den Broeke et al., 2013; Vuckovic et al., 2014; González-Roldán et al., 2016; Zebhauser et al., 2023). However, existing studies in this cohort are relatively few in number and the studies published so far have focused on the signals detected in either healthy participants exposed to acute experimental pain or in patients suffering from acute pain (Mouraux and Iannetti, 2018; Reckziegel et al., 2019). Thus, observed results mainly apply to a single type of condition at a certain point in time, which could easily be confounded with signals responsible for a long-lasting condition, such as chronic pain.

In contrast to acute pain, chronic pain involves complex peripheral and supraspinal brain mechanisms, where details on the underlying mechanisms remain incompletely known (von Hehn et al., 2012; Sun et al., 2021). Because of the multidimensional nature of chronic pain, a biomarker specific to the pathology has the potential to serve more than one purpose, thus being multifaceted and combinatorial (Ploner et al., 2017; Levitt and Saab, 2019). An overview of the studies included in this review, together with a summary of their representative features and biomarker type for each pain disease, can be seen in Tables 1–3.

### 5.1. Diagnostic biomarkers

A diagnostic biomarker indicates the presence of a condition or disease, like chronic pain. Most studies on chronic pain fall under this category (Van Der Miesen et al., 2019), primarily those associating chronic pain with group differences in EEG features (Reckziegel et al., 2019).

Studies assessing the potential of EEG as a diagnostic biomarker for chronic pain have emerged in the past decade and have led to the discovery of specific brain regions where relevant EEG changes associated with chronic pain are commonly observed. As presented in Table 1, the majority of the earlier studies recorded rs-EEG potentials in a few subjects. Nevertheless, they demonstrated promising findings indicating changes in specific frequency bands within targeted structures (De Vries et al., 2013; van den Broeke et al., 2013; Vuckovic et al., 2014; Navarro López et al., 2015; González-Roldán et al., 2016; Meneses et al., 2016). The described observations, mainly localized in the frontal, parietal, and occipital cortices, include both enhanced and reduced peak alpha frequency and theta responses, increased beta-band power, and increased ERD in the same bands—suggesting their potential as diagnostic biomarkers for chronic pain (De Vries et al., 2013; van den Broeke et al., 2013; Sufianov et al., 2014; Vuckovic et al., 2014; Navarro López et al., 2015; González-Roldán et al., 2016; Camfferman et al., 2017) (please see Table 2).

**Table 2:** Representative features and their biomarker types for each pain disease reviewed. Pain disease columns are sorted by descending quantity of papers reviewed; rows are sorted by descending count of observations. Format: “*Potential biomarker* (count of shared observations/number of papers reviewed in that pain disease; biomarker type(s)) [Reference(s)].”

Fibromyalgia	Chronic pain	Pancreatitis	Central neuropathic pain	Hip pain	Failed back surgery syndrome	Peripheral neuropathy	Low back pain	Diabetic polyneuropathy (DPN)
Decreased alpha band power (5/18; diagnostic, predictive) (Sufianov et al., 2014; González-Roldán et al., 2016; Meneses et al., 2016; Lancaster et al., 2017; Ferdek et al., 2019)	Decreased alpha band power (2/8; diagnostic) (Vachon-Presseau et al., 2016; Telkes et al., 2020)	Increased theta band power (2/5; monitoring) (Graversen et al., 2012; Jensen et al., 2013)	Alpha band activity (1/4; prognostic) (Vuckovic et al., 2018)	Frontal delta power (1/3; predictive) (Yüksel et al., 2019)	Decreased peak alpha frequency (2/3; diagnostic, monitoring) (Baliki et al., 2012; Uygur-Kucukseymen et al., 2020)	Decreased alpha band power (1/3; monitoring) (Prinsloo et al., 2017)	Decreased alpha band power (2/3; diagnostic, monitoring) (Gram et al., 2017; Baroni et al., 2020)	Increased theta band connectivity (1/2; diagnostic) (Martín-Brufau et al., 2021)
Decreased theta band power (3/18; diagnostic) (Sufianov et al., 2014; Meneses et al., 2016; Zortea et al., 2021)	Increased theta band connectivity (1/8; diagnostic) (Schulz et al., 2012a,b)	Decreased peak alpha frequency (1/5; diagnostic) (De Vries et al., 2013)	Increased ERD in alpha band (1/4; diagnostic) (Vuckovic et al., 2014)	Frontal spectral power between 1 and 35 Hz (1/3; monitoring) (Liu et al., 2015)	Increased beta band power (1/3; diagnostic, monitoring) (Baliki et al., 2012; Uygur-Kucukseymen et al., 2020)	Increased beta band power (1/3; monitoring) (Prinsloo et al., 2017)	Delta and theta bands correlated with self-reported pain intensities (1/3; diagnostic) (Parker et al., 2021)	Increased alpha band connectivity (1/2; diagnostic) (Martín-Brufau et al., 2021)
Increased theta band power (3/18; diagnostic) (González-Roldán et al., 2016; May et al., 2021)	Increased gamma band connectivity (1/8; diagnostic) (Dinh et al., 2019)	Increased beta band power (1/5; diagnostic, monitoring) (Jensen et al., 2013)	Increased ERD in theta band (1/4; diagnostic) (Vuckovic et al., 2014)	Increased frontal connectivity (1/3; diagnostic) (Jensen et al., 2021)	Decreased beta-2 band power (1/3; monitoring) (Uygur-Kucukseymen et al., 2020)	Low-beta band activity (1/3; diagnostic) (Kimura et al., 2021)		
Increased beta band power (3/18; diagnostic) (Sufianov et al., 2014; Meneses et al., 2016; May et al., 2021)	Over-activation in frontal and parietal areas (1/8; diagnostic, monitoring) (Santana et al., 2021)	Decreased alpha/beta power ratio (1/5; diagnostic, monitoring) (Jensen et al., 2013)	Increased ERD in beta band (1/4; diagnostic) (Vuckovic et al., 2014)					
Decreased alpha-2 band power (1/18; diagnostic) (Villafaina et al., 2019)	Increased theta band power (1/8; monitoring) (Schouppe et al., 2020)							
Increased beta band connectivity (1/18; diagnostic) (Zhou et al., 2018)	Decreased presence of microstate D (1/8; diagnostic) (Bernardi et al., 2021)							
Decreased beta band power (1/18; diagnostic, predictive) (Lancaster et al., 2017)	Increased theta global network efficiency (1/8; monitoring) (Teixeira et al., 2021)							
Decreased SMR/theta power ratio with neuro-feedback therapy (1/18; monitoring) (Di Pietro et al., 2018)								

Migraine	Orofacial pain	Post-concussive pain	Postherpetic neuralgia	Trigeminal neuropathy	Endometriosis	Lumbar back pain	Musculoskeletal pain	Post-mastectomy pain	Rheumatoid arthritis
Greater cortical coherence during baseline (1/2; diagnostic, prognostic) (Mendonça-de-Souza et al., 2012)	Decreased frontal and central alpha band power (1/2; diagnostic) (Wei et al., 2022)	Increased delta band power (1/2; diagnostic) (Lendaro et al., 2021)	Central-parietal beta band spectral entropy (1/2; predictive) (Heitmann et al., 2022)	Increased theta band power (1/2; diagnostic) (de Melo et al., 2020)	Increased beta connectivity in frontal, central, and temporal areas (1/1; diagnostic) (Lee et al., 2021)	Low-gamma band activity (1/1; diagnostic) (Teel et al., 2022)	Theta band permutation entropy (1/1; diagnostic) (Camfferman et al., 2017)	Enhanced alpha band power in parietal and occipital areas (1/1; diagnostic) (van den Broeke et al., 2013)	Increased alpha band power (1/1; diagnostic) (Cao et al., 2018)
Higher prefrontal complexity in the preictal phase (1/2; prognostic) (Thibaut et al., 2017)	Increased frontal and central gamma band power (1/2; diagnostic) (Wei et al., 2022)	Increased theta band power (1/2; diagnostic) (Lendaro et al., 2021)	Increased prefrontal gamma band power (1/2; diagnostic, monitoring) (Barbosa-Torres and Cubo-Delgado, 2021)						

**Table 3:** Representative features pooled across all pain disease types. Similar features are combined within the same row in column 1.

Potential biomarker	Biomarker type (s)	Number of claims^a^ (64 total features)
Decreased alpha band power; Decreased alpha-2 band power; Decreased peak alpha frequency; Increased ERD in alpha band; Decreased frontal and central alpha band power	Diagnostic, Predictive, Monitoring	16
Increased theta band power; Increased theta band connectivity; Theta band correlated with self-reported pain intensities	Diagnostic	11
Increased beta band power; Increased beta band connectivity; Increased beta connectivity in frontal, central, and temporal areas	Diagnostic, Monitoring	8
Decreased theta band power; Increased ERD in theta band	Diagnostic	4
Decreased beta band power; Increased ERD in beta band; Decreased beta-2 band power	Diagnostic, Predictive	3
Increased prefrontal gamma band power; Increased gamma band connectivity; Increased frontal and central gamma band power	Diagnostic, Monitoring	3
Increased alpha band power; Increased alpha band connectivity; Enhanced alpha band power in parietal and occipital areas	Diagnostic	3
Increased delta band power; Delta band correlated with self-reported pain intensities	Diagnostic	2

Rows are sorted by descending number of observations. Decreased alpha power prevails among all other potential biomarkers, accounting for 25% of all those reviewed.

a For brevity, only showing potential biomarkers with more than one observation; fourteen (14) potential biomarkers are omitted, which can be found in Table 1.

In one of the largest studies to date, Dinh et al. used SVM to demonstrate increased connectivity at theta (4–8 Hz) and gamma (50–100 Hz) frequencies in frontal regions, as well as global network reorganization (Dinh et al., 2019). Moreover, they demonstrated a decreased global efficiency at gamma frequencies in chronic pain patients. Such patterns have previously demonstrated involvement in the pathophysiology of chronic pain and are now better investigated. However, as described in Table 2, there still seems to be a continued discrepancy in the reported power responses in the theta, alpha, and beta bands, complicating the proposal of a consistent and reliable biomarker for chronic pain (Freye and Levy, 2006; Navarro López et al., 2015; Martín-Brufau et al., 2021). Moreover, the decoder performed only at 57% accuracy—close to chance-level, leaving much room for improvement. In more recent years, researchers’ primary goal in improving decoding performance has been motivated primarily by the goal of optimizing model generalization, where the application of SVM classifiers has led to an improvement in accuracy with up to 93.7% (Misra et al., 2017; Kragel et al., 2018; Levitt et al., 2020; Buchanan et al., 2021; Lendaro et al., 2021; Zolezzi et al., 2021; Teel et al., 2022; Topaz et al., 2022).

With gradual improvements in the ML algorithms over the years, there is a trend of testing their applicability in clinical practice, especially for diagnostic, monitoring, and prognostic purposes in the context of chronic pain (Mendonça-de-Souza et al., 2012; Sufianov et al., 2014). These applications would allow us to detect individuals with an increased risk of developing a certain condition, for example, the transition from acute to chronic pain. More importantly, it allows us to track the trajectory of pain development after applying certain therapies. This stratification of patients could serve to guide and inform future treatment and adds an additional quantitative objective measure of pain (Thibaut et al., 2017; Ahn et al., 2019; Yüksel et al., 2019; Santana et al., 2021; Heitmann et al., 2022).

As an example, and as a continuation of previous studies, Vuckovic et al. (2022) developed and further trained their own classifiers to evaluate subjects with central neuropathic pain. They provided evidence for the potential of utilizing non-oscillatory, non-linear features of EEG not only as a diagnostic biomarker but also for prognostic purposes. Thus, their study suggests that ML models can be trained not only to determine the presence of pain but also to predict the delay after which patients start showing symptoms of pain-state transition. However, pain is a highly subjective experience and often presents itself heterogeneously. Studies so far have not been able to present a diagnostic biomarker with enough validation and generalizability for clinical settings.

The extraction of spatial patterns and the detection of changes in oscillations have moved the field one step closer to producing a diagnostic biomarker for the presence of chronic pain. We now know that a distributed network of cortical circuits regulates pain with knowledge of specific regions involved in pain processing—the S1, ACC, and insular cortex (Liberati et al., 2018; Van Der Miesen et al., 2019; Sun et al., 2021). Furthermore, we know that noxious stimulation can evoke neural responses from these regions, like changes in theta and high gamma power (Baliki et al., 2012; Liu et al., 2015; Ploner et al., 2017; Zhang et al., 2017; Prichep et al., 2018; Schouppe et al., 2020). Currently, these features are described primarily in studies of acute experimental pain—with relatively few on chronic pain. Hence, to propose a diagnostic biomarker of chronic pain, further studies are needed to identify and confirm discriminative features specific to chronic pain states.

### 5.2. Prognostic biomarkers

Prognostic biomarkers serve to identify the likelihood of developing a disease or state, to track the progression or recurrence of a disease, or to identify the transition from one disease state to another, e.g., from an acute to chronic pain state (Baliki et al., 2012; Tracey et al., 2019; Van Der Miesen et al., 2019).

In general, difficulties have been noted in the studies trying to propose the usage of EEG as a prognostic biomarker. The main concern arises from the complex, dynamic nature of pain which limits the ability to capture EEG signals of prognostic value, requiring EEG recordings over a longer period. Nonetheless, there is a possibility that features learned through ML in studies using other neuroimaging modalities, such as fMRI (Baliki et al., 2012), can be used to monitor the transition between disease states from continuous EEG signals to be used as monitoring biomarkers. Meanwhile, only a few studies have examined this possibility in a longitudinal cohort of pain patients.

In 2018, Vuckovic et al. (2022) demonstrated the potential utility of EEG as a prognostic biomarker after using previous datasets with previously recorded EEG signals in patients with painful and non-painful central neuropathic pain. By testing three classifiers (artificial neural network, SVM, and linear discriminant analysis) on EEG band power in resting state data recorded over time, they demonstrated that a transferable learning classifier learning classifier could detect patients at risk for developing painful chronic neuropathic pain with 86% accuracy. The study also suggested that it is possible to further develop and expand the purpose of a biomarker by using already existing data sets (Vuckovic et al., 2014).

To our knowledge, there are only a handful of published studies using EEG as a potential biomarker solely for prognostic purposes in chronic pain patients. Due to its complex nature and clinical importance, a prognostic biomarker for chronic pain requires rigorous model training and validation with large data sets to achieve high sensitivity and high specificity, as well as good generalizability. With an increasing number of studies on diagnostic and monitoring biomarkers, studies investigating its prognostic counterpart are likely to increase over the coming years, potentially relying on existing data from the diagnostic and monitoring arm of the field (Vachon-Presseau et al., 2016).

### 5.3. Predictive and monitoring biomarkers

Available knowledge of neural processes and pathways associated with the presence of pain has initiated the development of ***predictive biomarkers** and **monitoring biomarkers*** for chronic pain. A ***predictive biomarker*** enables the identification of individuals who are more likely to respond to certain therapies than individuals without the biomarker (Reckziegel et al., 2019; Tracey et al., 2019; Van Der Miesen et al., 2019; Eldabe et al., 2022). A ***monitoring biomarker***, on the other hand, helps to serially measure the effect of an intervention or therapy. By combining both, we could predict a patient’s response to a certain therapy, enabling the development of a customized intervention program ahead of time.

For decades, scientists have explored the clinical implication of EEG as a monitoring biomarker for chronic pain. This has been done mainly by evaluating EEG signals before and after applying certain therapies—either individually or combined across subjects. Examples of such interventions include acupuncture, analgesics and anticonvulsants, epidural cord stimulation, neurofeedback treatment, surgical treatment, and transcranial stimulation therapies (Graversen et al., 2012; Jensen et al., 2013; Sufianov et al., 2014; Prinsloo et al., 2017; Thibaut et al., 2017; Ahn et al., 2019; Yüksel et al., 2019; de Melo et al., 2020; Barbosa-Torres and Cubo-Delgado, 2021; Heitmann et al., 2022).

As presented in Table 1, only a few studies attempting to assess EEG changes after applying targeted therapies have used healthy controls for comparison, hampering the predictive potentials of the proposed biomarker (Gram et al., 2017; Prinsloo et al., 2017; Ahn et al., 2019; de Melo et al., 2020; Lee et al., 2021; Zortea et al., 2021; Wei et al., 2022).

Notably, the majority of studies evaluating potential therapies have observed noticeable, statistically significant differences in the powers of theta, alpha, beta, and gamma activity in regions associated with chronic pain states (Zhou et al., 2018; de Melo et al., 2020; Lee et al., 2021; Patel et al., 2021; Zortea et al., 2021). These findings further strengthen the potential of EEG as a monitoring biomarker. With data over longer periods, from both healthy controls and chronic pain patients, the development of a robust composite biomarker serving diagnostic, predictive, prognostic, and monitoring purposes will be more readily achievable.

## 6. The future of EEG as a biomarker for chronic pain

In the last decade, the EEG pain research field has seen rapid progress. Though as we look into the future, we predict collaborative efforts will be crucial for achieving the development of any EEG biomarker for pain, especially one that is composite. Below we list some recommendations to push the EEG pain research community forward.

Data sharing and data pooling across study groups have proven to be appealing and perhaps essential methods to address the need for large data sets for ML analysis of EEG recordings (Van Der Miesen et al., 2019; Davis et al., 2020). Research groups may test various features and ML methods on the same large dataset, making for more robust comparisons between models, and facilitating faster discovery of potential features toward a composite biomarker for chronic pain. Moreover, by pooling data across various study groups, the validity of a potential biomarker would increase as the sample size gets larger, allowing for more robust cross-validation (Davis et al., 2020). In addition, the transparency of a potential EEG biomarker would be significantly improved by homogenized reporting standards. Currently, there exists a great discrepancy between reported results, and as part of an effort to improve future outcomes, new reporting guidelines have been developed and presented (Mari et al., 2022). However, large, multi-center datasets do come with a caveat: increased subject variability may hinder the clarity of cross-subject predictors.

Another benefit of data pooling is the diversity of chronic pain conditions, making a potential biomarker more generalizable. Due to slight variations in population samples, two research groups studying the same pain disease with similar experimental and analysis techniques could arrive at contrasting results. For example, a number of the studies in this review are focused on one specific pain disease cohort: fibromyalgia patients, a condition that is much more common in women than in men, creating an obstacle for a generalizable biomarker. Thus, data pooling has the potential to reduce the impact of sample population variations in small datasets by increasing the diversity in multi-center datasets.

Another innovative approach that has emerged recently is to combine rs-EEG studies with stimulus-evoked signals (Table 1). Pain is a dynamic process, and chronic pain involves both tonic and phasic components. Thus, by analyzing both resting-state and stimulus-evoked EEG potentials, we can further reduce the risk of confounders and improve sensitivity and specificity (Hansen et al., 2017).

Furthermore, Ávila et al. published an open and fully automated pipeline (DISCOVER-EEG) enabling easy, fast, and homogenous preprocessing, analysis, and visualization of rs-EEG data. This is an important step forward and should be taken as an example for future studies—where a tool like this will most likely promote open and reproducible research on brain function (Gil Avila et al., 2023). Optimally, this tool could be further developed into an unsupervised or semi-supervised ML method, allowing us to use largely unlabeled data, which would increase the generalizability of the potential biomarker itself. Moreover, standardized ML processes could contribute to the use of good ML practices, where commonly noted mistakes in current studies are results that may reflect overfitting or other anomalies in the ML implementation. Importantly, future studies also need to focus on diversity, equity, and inclusion in both training and testing datasets to further ensure good ML practice.

Advances in EEG source localization may also help improve the validity and reliability of a potential EEG biomarker for pain. Specifically, these methods could be used to inform which scalp electrodes are best positioned to record pain-associated brain activity, improving biomarker transparency and usability. As an example, the study conducted by Cao et al. demonstrated that using just a handful of leads may be enough to detect the presence of a disease, making future studies easier to conduct, but also facilitating continuous and longer EEG recording for monitoring purposes (Cao et al., 2018). This would be further facilitated by way of a portable device (Pu et al., 2021; Eldabe et al., 2022).

### 6.1. The potential for multimodal biomarkers

Studies on multimodal biomarkers are emerging for chronic pain patients (Prichep et al., 2018; Tracey et al., 2019; Eldabe et al., 2022). The incorporation of computational methods to conventional neurophysiological techniques such as EEG can be combined with other testing modalities, such as clinical reports, blood biomarkers, and quantitative sensory testing, to quantify pain and to predict outcomes for chronic pain patients (Califf, 2018; Mari et al., 2022).

Over recent years, a shift in paradigm for decoding chronic pain has already occurred, by incorporating objective neural signals into more subjective measurements of pain such as pain and mood questionnaires, as well as physiological data such as pulse and skin conductance measurements (Lancaster et al., 2017). With our current understanding of the EEG patterns associated with chronic pain states, and with the continuous improvement of ML algorithms, we now have the tools to propose multimodal biomarkers in the future. Another development in biomarker research is that an appropriately collected and curated database could be applied to the development of multiple biomarkers serving more than one clinical purpose (Vuckovic et al., 2014, 2018).

### 6.2. Limitations of this review

As a narrative review, we did not employ a systematic set of criteria for study inclusion, in part due to the relatively disparate literature in machine learning and EEG studies in pain. By presenting concepts from the perspective of clinical applicability, we aimed to facilitate an understanding of the application of EEG and machine learning in studies of chronic pain without the use of restrictive language.

### 6.3. Summary

In summary, impactful studies have been conducted in the past decade showing the potential for an EEG-based biomarker for chronic pain. Through the establishment of standardized practices and improved collaborations between members of the field, EEG-based techniques have the potential to become a key component of chronic pain diagnosis and treatment.

## Author contributions

MR wrote the first draft of the manuscript. GK, LD, ZC, and JW wrote sections of the manuscript. All authors contributed to the article and approved the submitted version.

## Conflict of interest

JW is a cofounder of Pallas Technologies, Inc., and ZC is a scientific advisor of Pallas Technologies, Inc. JW and ZC are inventors of a pending US patent application of pain treatment technology.

The remaining authors declare that the research was conducted in the absence of any commercial or financial relationships that could be construed as a potential conflict of interest.

## Publisher’s note

All claims expressed in this article are solely those of the authors and do not necessarily represent those of their affiliated organizations, or those of the publisher, the editors and the reviewers. Any product that may be evaluated in this article, or claim that may be made by its manufacturer, is not guaranteed or endorsed by the publisher.
